# Adrenergic Activation of Melatonin Secretion in Ovine Pineal Explants in Short-Term Superfusion Culture Occurs via Protein Synthesis Independent and Dependent Phenomena

**DOI:** 10.1155/2014/715708

**Published:** 2014-07-15

**Authors:** Bogdan Lewczuk, Natalia Ziółkowska, Magdalena Prusik, Barbara Przybylska-Gornowicz

**Affiliations:** Department of Histology and Embryology, Faculty of Veterinary Medicine, University of Warmia and Mazury, Oczapowskiego Street 13, 10-719 Olsztyn, Poland

## Abstract

The ovine pineal is generally considered as an interesting model for the study on adrenergic regulation of melatonin secretion due to some functional similarities with this gland in the human. The present investigations, performed in the superfusion culture of pineal explants, demonstrated that the norepinephrine-induced elevation of melatonin secretion in ovine pinealocytes comprised of two subsequent periods: a rapid increase phase and a slow increase phase. The first one included the quick rise in release of N-acetylserotonin and melatonin, occurring parallel to elevation of NE concentration in the medium surrounding explants. This rapid increase phase was not affected by inhibition of translation. The second, slow increase phase began after NE level had reached the maximum concentration in the culture medium and lasted about two hours. It was completely abolished by the treatment with translation inhibitors. The obtained results showed for the first time that the regulation of N-acetylserotonin synthesis in pinealocytes of some species like the sheep involves the on/off mechanism, which is completely independent of protein synthesis and works very fast. They provided strong evidence pointing to the need of revision of the current opinion that arylalkylamines N-acetyltransferase activity in pinealocytes is controlled exclusively by changes in enzyme abundance.

## 1. Introduction

The diurnal rhythmicity, manifested in the majority of metabolic processes occurring in cells, tissues, and organs, has its source in adaption of organisms to cyclic successions of day and night. In mammals, the photoperiodic time measurement system consists of the retina with light-sensitive, melanopsin-containing ganglion cells [1, 2], the hypothalamic suprachiasmatic nucleus (SCN) with neurons showing spontaneous, circadian expression of clock genes [3, 4], and the pineal gland with pinealocytes transducing the neuronal signal from SCN into the hormonal code-melatonin [4, 5]. The diurnal rhythm of melatonin secretion, with elevated level at night, provides all cells of the body with information about the time of day and the season [5].

In the mammalian pineal gland, the rhythmic secretion of melatonin synthesis is driven by norepinephrine (NE) released from the sympathetic nerve fibers [6]. These fibers constitute the last part of multisynaptic pathway connecting the gland with SCN and indirectly with the retina [7]. Several studies performed during the last 25 years of the 20th century on the rat pineal gland enable the scientists to prepare a detailed model of adrenergic regulation of melatonin secretion in this species [4, 8], which has been successively updated [9–13]. This model explains very well almost all phenomena related to the synthesis and secretion of melatonin in the rat.

The activation of melatonin secretion in rat pinealocytes occurs via stimulation of *β*_1_- and *α*_1_-adrenoceptors [8, 14]. The binding of NE to *β*_1_-adrenoceptors leads to elevation of cAMP level and activation of protein kinase A (PKA), which, among other substrates, phosphorylates the CRE-binding protein (CREB) [8, 15, 16]. Phospho-CREB increases transcription of CRE-containing genes including arylalkylamine N-acetyltransferase (AA-NAT) gene and inducible cAMP early repressor (ICER) gene [8, 17]. As a consequence, the content and activity of AA-NAT, an enzyme catalyzing conversion of serotonin into N-acetylserotonin, increase several folds, but rather slowly, during the first part of the night [17, 18]. Secretion of melatonin rises parallel to elevation of AA-NAT activity; however, at the peak, it is limited by acetylserotonin methyltransferase (ASMT) activity [9]. The AA-NAT protein is shielded from degradation, which may occur due to proteasomal proteolysis, by PKA-mediated phosphorylation and binding with 14-3-3 protein [19]. This protection of AA-NAT is turned off after light-exposition, which causes drop in cAMP level and PKA activity [4, 10]. The transcription of AA-NAT gene decreases gradually during the second part of the night due to inhibition by ICER [16, 18]. Moreover, NE activates *α*_1_-adrenoceptors, which in turn potentiates the effect of *β*_1_-adrenoceptors stimulation [14].

Initially, the rat model of pineal activity regulation was considered as valid for all mammalian species. However, the studies of ovine and bovine pineals demonstrated the occurrence of fundamental differences in the regulation of melatonin synthesis among investigated rodents and ruminants [20–24]. In the sheep pineal gland, AA-NAT mRNA levels only slightly differ between day and night, despite marked diurnal variations in AA-NAT activity [23]. Moreover, the AA-NAT mRNA level was not increased in ovine pinealocytes by stimulation with forskolin [24]. Similarly, the adrenergic stimulation of bovine pinealocytes in monolayer culture did not change the level of AA-NAT mRNA but markedly increased the content and activity of this enzyme [20]. The increase in AA-NAT protein content and activity was noted after the treatment with inhibitors of proteasomal proteolysis; therefore, the degradation of AA-NAT was proposed as the main process responsible for regulation of melatonin secretion in the bovine [20]. It is generally accepted that AA-NAT is constitutively produced in ruminant pinealocytes and it is degraded during photophase by proteolysis [20, 21]. At night, when the enzyme is protected from degradation, it accumulates in cells and melatonin secretion increases.

Subsequent studies showed that the rat model of adrenergic regulation of melatonin secretion is also not useful for primates including the human [25–27]. In* Macaca mulatta*, no differences in AA-NAT mRNA levels were observed between day and night, while the enzyme protein content and activity were tenfold higher at nighttime when compared with daytime [25]. The similarity of results obtained in ruminants and primates pointed to the ovine pineal gland as potentially useful model for the studies on regulation of melatonin secretion in the human [26, 27]. The investigations performed in the degu have shown that significant differences in regulation of the pineal melatonin synthesis appear even within the order Rodentia [28].

Both in the rat and the ruminant models of adrenergic regulation of melatonin secretion, it is currently assumed that the regulation of AA-NAT activity occurs via changes in the enzyme content.

The aim of the study was to characterize (1) the time-course of changes in N-acetylserotonin and melatonin release from ovine pineal explants during the adrenergic stimulation and (2) the effect of transcription inhibitor, actinomycin D (ACT D), and translation inhibitors, cycloheximide (CLX) and anisomycin (ANS), on NE-induced increase in melatonin secretion. For comparative purposes, rat pineals were also used in the study. The present study demonstrates for the first time that acetylation of serotonin in pinealocytes may be switched on and off without involving the protein synthesis and this mechanism is responsible for a rapid increase in melatonin secretion in the ovine pineal. Our results and the data from the studies of ovine and human AA-NAT* in vitro* and in transfected nonpineal cells [29–32] provide strong arguments against the thesis that intensity of serotonin acetylation is controlled exclusively by synthesis and degradation of AA-NAT.

## 2. Material and Methods

### 2.1. Chemicals

Medium 199 containing Earle's salt and HEPES (SIGMA, USA) was prepared from a powder according the manufacturer instruction (pH adjusted to 7.2 with NaOH) and sterilized by filtration. Before use, the sterile solution of ascorbic acid was added to the medium to give a final concentration of 30 mg per 100 mL. Eagle's minimum essential medium (MEM) without leucine was provided by Life Technologies (USA). Antimelatonin antibody G/S/704-6483 was purchased from Stockgrand Ltd, University of Surrey, Great Britain. ^3^H-melatonin (87 Ci/mM) and ^3^H-leucine (140 Ci/mM) were from PerinElmer Radiochemical (USA). Methanol of gradient-grade HPLC purity was provided by Merck Millipore (Germany). Sodium acetate and disodium EDTA of the highest chemical purity were from J. T. Baker Chemicals (Germany). All other chemicals were purchased from Sigma-Aldrich (USA). Ultrapure water (18,2 MΩ, TOC ≤ 5 ppb), freshly prepared by Milli-Q integral purification system (Merck Millipore, Germany), was used in all procedures.

### 2.2. Animals and Tissues

Females of the Polish Lowland sheep were raised under natural light conditions up to age of three months. At least two weeks before experiments, they were transported to the animal laboratory rooms and kept under a cycle of 12 hours light : 12 hours dark, with a photophase from 7.00 a.m. to 7.00 p.m. (fluorescent lighting of 500 lux intensity at the floor level). The animals were slaughtered between 02.00 and 03.00 p.m.

Female Wistar rats, aged 4 months, were obtained from a local breeding population. They were kept under a cycle of 12 hours light : 12 hours dark (photophase from 7.00 a.m. to 7.00 p.m., with fluorescent lighting of 500 lux intensity at the cage floor level). The rats were anesthetized using isoflurane vapors (Iso-Vet, Primal Healthcare Ltd., UK) and killed by decapitation (between 02.00 and 02.30 p.m.).

The ovine (*n* = 26) and rat (*n* = 30) pineal glands were immediately removed, placed in the culture medium, and transported within three minutes to the cell culture laboratory. The study was carried out in a strict accordance with Polish law of animal welfare.

### 2.3. Superfusion Culture

The ovine pineal glands were divided under sterile conditions into three or four explants, which were randomly assigned to control and experimental groups. The explants of ovine pineals or the entire rat pineals were covered with a nylon mesh and placed into culture chambers (volume 0.5 mL). The lower pool of each chamber was connected via a system of tubes and valves to two or three containers with the culture medium, continuously gassed with a mixture of 5% CO_2_ and 95% O_2_. The upper pool of the culture chamber was attached to a multichannel peristaltic pump and a manual fraction collector. The incubation of explants was performed at 38.5°C (the sheep pineal explants) or 37°C (the rat pineals), at a flow rate of 0.2 mL/min (experiments I–IV) or 0.05 mL/min (experiments V-VI). The medium fractions were collected every 5 or 10 min and frozen at −75°C until assays.

### 2.4. Experimental Procedures

**Figure 1:**
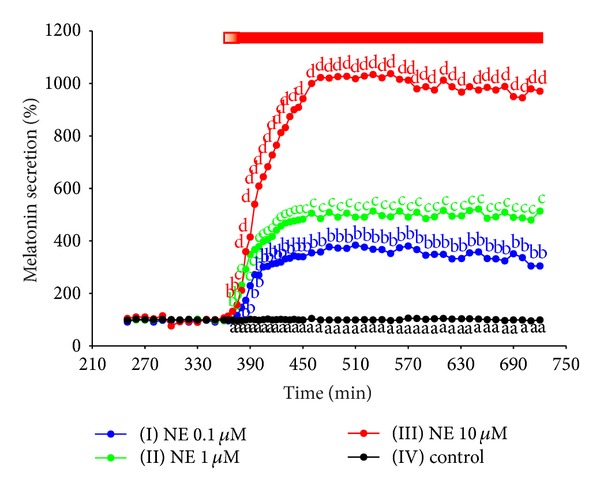
Experiment I. Effect of different concentrations of NE on melatonin secretion from the ovine pineal explants. The ovine pineal glands (*n* = 4) were divided into four explants, which were randomly assigned to the control and experimental groups. The explants were incubated between 361 and 720 min of the experiment in the medium containing group I, 0.1 *μ*M NE; group II, 1 *μ*M NE; and group III, 10 *μ*M NE. The explants from group IV (control) were not treated with NE. The mean melatonin secretion between 301 min and 360 min of the experiment was taken as 100%. The data presented are means. The values significantly different (*P* ≤ 0.05) between groups were signed with different letters. Red bar is period of incubation in the medium with NE.

**Figure 2:**
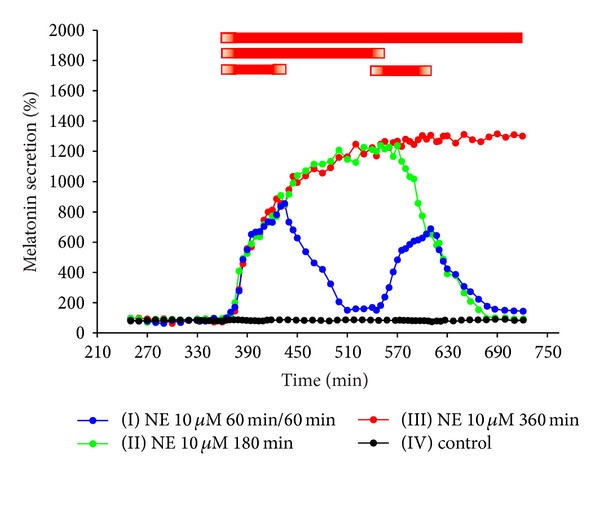
Experiment II. Time-course of NE-evoked changes in melatonin secretion from the ovine pineal explants. The ovine pineal glands (*n* = 4) were divided into four explants, which were randomly assigned to the control and experimental groups. The explants were treated with NE as follows: group I, from 361 to 420 min and from 541 to 600 min of the experiment; group II, between 361 and 540 min of the experiment; and group III, between 361 and 720 min of the experiment. The explants from group IV (control) were not treated with NE. The mean melatonin secretion between 301 min and 360 min of the experiment was taken as 100%. The data presented are means. Red bars are periods of incubation in the medium with NE.

**Figure 3:**
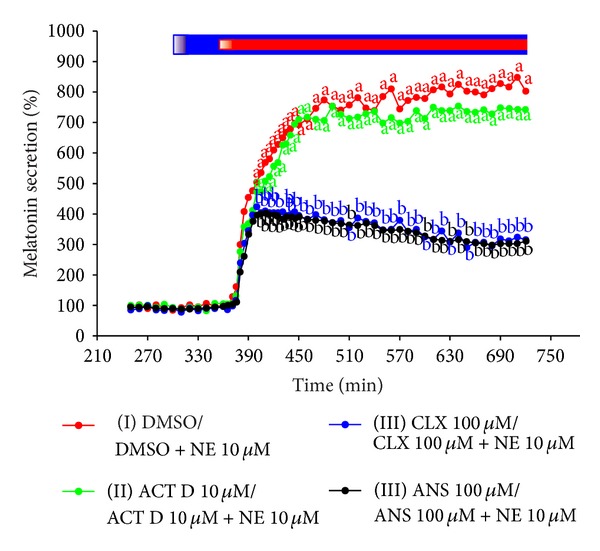
Experiment III. Effect of NE on melatonin secretion from the ovine pineal explants treated with transcription and translation inhibitors. The ovine pineal glands (*n* = 4) were divided into four explants, which were randomly assigned to the experimental groups. The explants were incubated in the medium containing group I, 0.5% DMSO from 301 min to 360 min and 10 *μ*M NE + 0.5% DMSO from 361 min to 720 min of the experiment; group II, 10 *μ*M ACT D from 301 min to 360 min and 10 *μ*M NE + 10 *μ*M ACT D from 361 min to 720 min of the experiment; group III, 100 *μ*M CLX from 301 min to 360 min and 10 *μ*M NE + 100 *μ*M CLX from 361 min to 720 min of the experiment; and group IV, 100 *μ*M ANS from 301 min to 360 min and 10 *μ*M NE + 100 *μ*M ANS from 361 min to 720 min of the experiment. The mean melatonin secretion between 241 min and 300 min of the experiment was taken as 100%. The data presented are means. The values significantly different (*P* ≤ 0.05) between groups were signed with different letters. Blue bar is period of incubation in the medium with inhibitors; red bar is period of incubation in the medium with NE.

**Figure 4:**
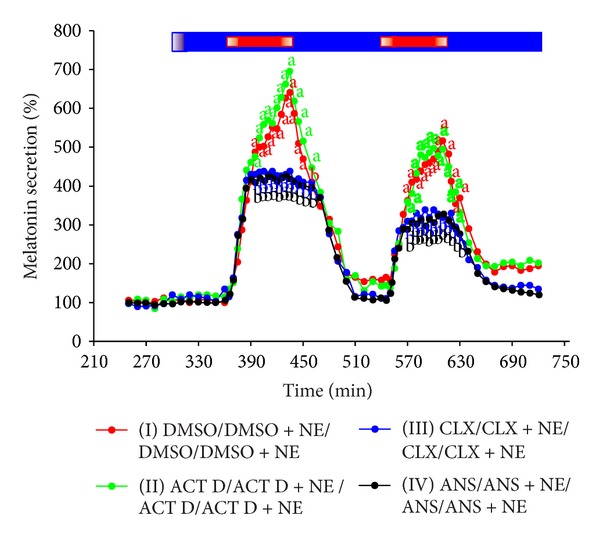
Experiment IV. Effect of repeated adrenergic stimulation on melatonin secretion from the ovine pineal explants treated with transcription and translation inhibitors. The ovine pineal glands (*n* = 4) were divided into four explants, which were randomly assigned to the experimental groups. The explants were incubated in the medium containing group I, 0.5% DMSO between 301 min and 720 min of the experiment; group II, 10 *μ*M ACT D between 301 min and 720 min of the experiment; group III, 100 *μ*M CLX between 301 min and 720 min of the experiment; and group IV, 100 *μ*M ANS between 301 min and 720 min of the experiment. The explants from all groups were treated two times with 10 *μ*M NE, from 360 min to 420 min and from 540 min and 600 min of the experiment. The mean melatonin secretion between 241 min and 300 min of the experiment was taken as 100%. The data presented are means. The values significantly different (*P* ≤ 0.05) between groups were signed with different letters. Blue bar is period of incubation in the medium with inhibitors; red bars are periods of incubation in the medium with NE.

**Figure 5:**
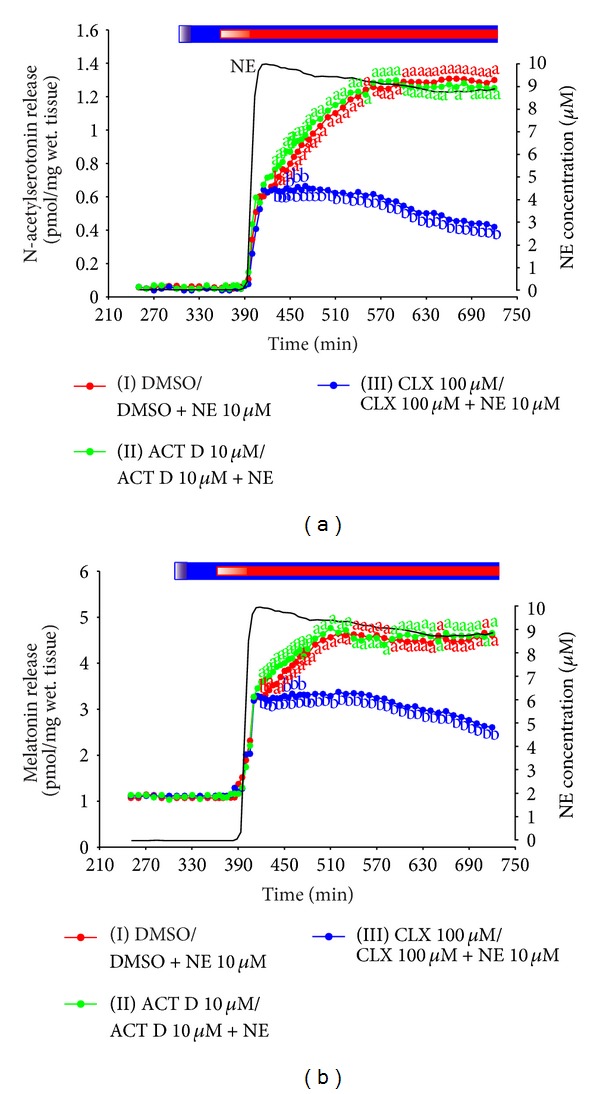
Experiment V. Effect of NE on N-acetylserotonin (a) and melatonin (b) release from the ovine pineal explants treated with transcription and translation inhibitors. The ovine pineal glands (*n* = 4) were divided into three explants, which were randomly assigned to the experimental groups. The explants were incubated in the medium containing group I, 0.5% DMSO from 301 min to 360 min and 10 *μ*M NE + 0.5% DMSO from 361 min to 720 min of the experiment; group II, 10 *μ*M ACT D from 301 min to 360 min and 10 *μ*M NE + 10 *μ*M ACT D from 361 min to 720 min of the experiment; and group III, 100 *μ*M CLX from 301 min to 360 min and 10 *μ*M NE + 100 *μ*M CLX from 361 min to 720 min of the experiment. The data presented are means. The values significantly different (*P* ≤ 0.05) between groups were signed with different letters. Black line is the level of NE in the culture medium. Blue bar is period of incubation in the medium with inhibitors; red bar is period of incubation in the medium with NE.

**Figure 6:**
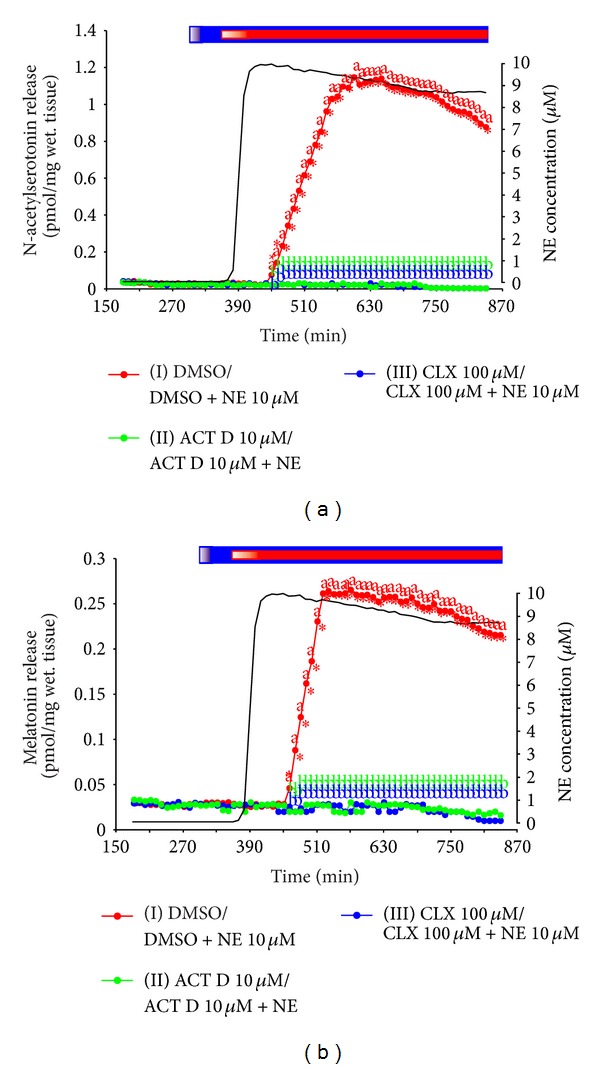
Experiment VI. Effect of NE on N-acetylserotonin (a) and melatonin (b) release from the rat pineal glands treated with transcription and translation inhibitors. The rat pineal glands (*n* = 12) were randomly assigned to the experimental groups. They were incubated in the medium containing group I, 0.5% DMSO from 301 min to 360 min and 10 *μ*M NE + 0.5% DMSO from 361 min to 840 min of the experiment; group II, 10 *μ*M ACT D from 301 min to 360 min and 10 *μ*M NE + 10 *μ*M ACT D from 361 min to 840 min of the experiment; and group III, 100 *μ*M CLX from 301 min to 360 min and 10 *μ*M NE + 100 *μ*M CLX from 361 min to 840 min of the experiment. The data presented are means. The values significantly different (*P* ≤ 0.05) between groups were signed with different letters. ∗Significantly different than in the same group between 241 and 300 min of the experiment. Black line is the level of NE in the culture medium. Blue bar is period of incubation in the medium with inhibitors; red bar is period of incubation in the medium with NE.

Experiments I–V were performed on the ovine pineal explants and experiment VI was conducted on the rat pineals. The detailed protocols of experiments were presented in legends of Figures 1–6.

### 2.5. Translation Measurement

The tissues were placed on meshes of organ culture inserts in appropriate 12-well culture plates containing 0.5 mL of MEM per well and incubated in a humidified atmosphere of 70 % O_2_ and 5% CO_2_ at 37°C (the rat pineal explants) or 38.5°C (the ovine pineal explants). After three hours of preincubation, the inserts with explants were transferred to the new culture plates containing the medium with 100 *μ*M CLX, 10 *μ*M ANS, or 0.5% DMSO and incubated for 60 min. Next, the explants were three times rinsed with MEM without leucine and transferred to the plates containing this medium with the addition of 370 kBq of ^3^H-leucine and 10 *μ*M NE + 100 *μ*M CLX, 10 *μ*M NE + 10 *μ*M ANS, or 10 *μ*M NE + 0.5% DSMO. The explants were incubated for 120 min, rinsed three times with MEM without leucine, and frozen at −75°C. The protein fraction was isolated and the radioactivity was measured as previously described [32].

### 2.6. Melatonin Radioimmunoassay

Melatonin concentration in the medium samples collected during the experiments I–IV was measured by a direct RIA with the use of G/S/704-6483 antibody and ^3^H-melatonin, according to the previously described and validated procedure [32]. The sensitivity of assay was 2.5 pg/tube. The intra- and interassay coefficients of variation were below 10%.

### 2.7. N-Acetylserotonin and Melatonin Assay by HPLC

N-acetylserotonin and melatonin concentrations in the medium samples from the experiments V and VI were measured using a gradient HPLC with fluorescence detection.

#### 2.7.1. Sample and Standards Preparation

Indolic compounds were dissolved in water, absolute methanol, or 0.1 M hydrochloric acid, prediluted in water, and finally diluted in medium 199. Samples or standard solutions were mixed with 1 M solution of perchloric acid in a proportion of 45 : 5, incubated in ice bath for 15 min, and centrifuged at 30,000 g for 20 min (Allegra 64R, Coulter Beckman, USA).

#### 2.7.2. Chromatographic Procedure

The assay was carried out with an Ultimate 3000 chromatographic system (Dionex, USA) which consisted of pump LPG 3400A with build-in four-channel degasser, autosampler WPS 3000SL, column thermostat TCC 3100, and fluorescence detector FLD 3400 RS. The injection volume of standards and samples was 100 *μ*L. The separation was performed on Hypersil Gold aQ column with 3 *μ*m particle size and dimensions of 150 × 4.6 mm, protected by a dedicated guard column (Thermo Scientific, USA) at a temperature of 30°C. The mobile phase was composed by online mixing of methanol and water solution of 5 mM sodium acetate and 0.01 mM disodium EDTA (pH = 4.5 by addition of acetic acid). The flow rate of mobile phase was 1 mL/min. The initial concentration of methanol was 5% (v/v). Next, between the 12th and 23rd min of the separation, the methanol content was linearly increased to the level of 30% (v/v) and then was kept at a constant value for 12 min. The methanol concentration was decreased to the initial level between the 35th and 40th min of the separation, and after 5 min long equilibration the system was ready to the next injection. The detection was performed at an excitation wavelength of 280 nm and an emission wavelength of 345 nm at a constant temperature of 45°C. The sensitivity of the detector was set to the maximum value of 8, with exception of two periods (from 0 to 4.25 min and from 8.50 to 10.00 min), when it decrease to the lowest values of 1. The system was controlled and the chromatograms were analyzed using Chromeleon 6.8 software (Dionex, USA). The method enabled the separation of 10 indoles and NE in medium 199. Limits of quantification for N-acetylserotonin and melatonin were 10 pg per injection and for NE 15 ng per injection.

### 2.8. Statistical Analysis

The data were analyzed using one-way ANOVA (experiments I–IV) or repeated-measures ANOVA (experiments V and VI) and the LSD test as a post hoc procedure using Statistica 10.0 software (StatSoft, USA).

## 3. Results

### 3.1. Experiments I–IV. Secretion of Melatonin from Ovine Pineal Explants

The treatment of ovine pineal explants with NE resulted in the statistically significant, concentration-dependent increase in melatonin secretion (Figure 1). The kinetic profiles of changes in melatonin release in response to the adrenergic stimulation showed a biphasic increase (Figures 1, 2, and 3). The first phase lasted about 20 min and included very rapid rise in the pineal hormone release. The melatonin secretion from the explants treated with 10 *μ*M of NE was about fivefold higher at the end of this phase than before the stimulation. During the second phase, the release of pineal hormone increased slowly, reaching the maximum level within 1.5–2 hours. The stimulation of explants with 10 *μ*M of NE elevated melatonin secretion to 800–1200% of the initial level. Then, the pineal hormone release remained at a stable, increased level as long as NE was present in the culture medium. The withdrawal of catecholamine from the medium resulted in stepwise decrease in melatonin secretion to the value before stimulation. The second adrenergic stimulation of ovine pineal explants caused slightly lower increase in melatonin secretion than the first treatment with NE (Figures 2 and 4).

The incubation of ovine pineal explants with ACT D did not influence the increase in melatonin secretion evoked by NE (Figures 3 and 4).

The adrenergic stimulation of ovine pineal explants incubated in the medium containing CLX (100 *μ*M) or ANS (10 *μ*M) resulted in the rapid, monophasic increase in melatonin release (Figures 3 and 4). The maximum level of secretion was noted after 20 min of the stimulation and it was similar to that observed at the same time in the explants treated solely with NE. Next, no further increase in melatonin release was noted in the groups of explants incubated with translation inhibitors. The pineal hormone secretion from these explants was stable for about one hour (Figures 3 and 4) and then it slowly decreased (Figure 3). As a consequence, after the initial phase of similar changes in melatonin secretion, the pineal hormone release from the explants incubated with translation inhibitors was significantly lower than that from the control explants (Figures 3 and 4). Despite some decline, melatonin secretion from the CLX and ANS treated explants was about threefold higher after six hours of the adrenergic stimulation than before exposition to NE (Figure 3). The second adrenergic stimulation of the CLX-treated explants also resulted in the significant elevation of melatonin secretion (Figure 4).

### 3.2. Experiment V. Release of N-Acetylserotonin and Melatonin from Ovine Pineal Explants

The treatment with NE at a concentration of 10 *μ*M induced the large, biphasic increase in N-acetylserotonin release from ovine pineal explants (Figure 5(a)). Initially, the release of this indoleamine increased parallel to the changes in NE content in the culture medium. During this period, lasting about 20 min, the level of N-acetylserotonin in the medium rose tenfold. Next, the release of N-acetylserotonin increased slowly and reached the maximum value, extending 20-fold the level before stimulation, after about three hours of the incubation with NE.

The changes in melatonin release in response to the adrenergic stimulation (Figure 5(b)) were similar to those found in the experiments I–IV presented above. The release of melatonin rose about threefold within the first 20 min of the NE-treatment. Later, the increase was slow and the maximum level of melatonin secretion, exceeding fivefold the value before stimulation, was reached after two hours of the stimulation.

The adrenergic stimulation of the explants pretreated with CLX resulted in the rapid, tenfold increase in the N-acetylserotonin release, which was parallel to the changes in NE level in the culture medium (Figure 5(a)). No differences in the release of N-acetylserotonin were noted between the CLX-treated and control explants during this period of time. The abovementioned increase was followed by a relatively stable release of the indoleamine. The slow phase of increase in N-acetylserotonin release, occurring in the control explants, was not observed in the CLX-treated explants. In consequence, the level of N-acetylserotonin release differed significantly between these groups of explants, starting from the end of the rapid response phase (Figure 5(a)).

The release of melatonin, like N-acetylserotonin, increased parallel to the changes in the content of NE in culture medium (Figure 5(b)). Within 20 minutes, the melatonin secretion was raised about threefold and reached the maximum level. The lack of further increase in melatonin secretion resulted in the statistically significant differences in the release of this indole between the CLX-treated and control explants.

The release of N-acetylserotonin was similar in the control explants and the explants treated with ACT D (Figure 5(b)). No differences were also noted in melatonin secretion between these groups of explants.

### 3.3. Experiment VI. Release of N-Acetylserotonin and Melatonin from Rat Pineals

For the first time, the significantly increased level of N-acetylserotonin in medium was noted 30 minutes after NE had reached the nominal concentration of 10 *μ*M in culture chambers (Figure 6(a)). The release of this indole from the rat pineals increased stepwise during the next two hours. Finally, it was about 40-fold higher than that before stimulation. The release of N-acetylserotonin remained at a stable, high level during three hours and then slightly decreased.

The melatonin secretion from the rat pineals increased parallel to the changes in N-acetylserotonin release; however, it reached the maximum level one hour before the peak of N-acetylserotonin (Figure 6(b)). The final phase of elevation of N-acetylserotonin release was not followed by the increase in melatonin secretion. The maximum level of melatonin secretion exceeded tenfold the value noted before the adrenergic stimulation.

The increase in release of N-acetylserotonin and melatonin in response to the adrenergic stimulation of the rat pineals was completely abolished by the treatment with ACT D and CLX (Figures 6(a) and 6(b)).

### 3.4. Inhibition of Translation by CLX and ANS in Ovine and Rat Pineal Explants

The incorporation of ^3^H-leucine into protein fraction of ovine pineal explants was reduced by more than 98% after the treatment with 100 *μ*M CLX and by more than 95% after the incubation with 10 *μ*M ANS (Table 1). The similar effects of CLX and ANS on ^3^H-leucine incorporation were observed in rat pineals (Table 1).

## 4. Discussion

According to our results, the adrenergic activation of melatonin secretion in ovine pineal explants comprises two subsequently occurring periods: the rapid increase phase and the slow increase phase. In the superfusion culture model, the first phase is represented by the rise in the pineal hormone secretion occurring parallel to the elevation of NE concentration in the medium surrounding explants. The melatonin release at the end of this phase is up to 5-6 times higher than that before the stimulation. These data demonstrate that ovine pinealocytes respond immediately to the adrenergic stimulation, with the several-fold increase in melatonin secretion. The initial, rapid elevation of secretory activity is followed by the slow increase phase, lasting up to two hours. In our* in vitro *model, the second phase of increase finally results in 10–12 times higher melatonin secretion (during treatment with 10 *μ*M of NE) than before the adrenergic stimulation.

The most important findings were obtained after the treatment of ovine pineal explants with inhibitors of translation and transcription. We demonstrated that the rapid phase of the NE-induced increase in melatonin secretion is completely independent of protein biosynthesis, while the slow phase requires translation of new proteins. The experiment with the repeated adrenergic stimulations showed that melatonin secretion can be at least two times switched on and off in the ovine pineal explants with inhibited protein synthesis. Both responses were restricted to the rapid phase of increase in melatonin secretion. It should be underlined that the efficiency of translation inhibitors used in our study was confirmed by testing ^3^H-leucine incorporation into proteins in pineal cells. The treatment of explants with ACT D showed that transcription does not play a significant role in the adrenergic activation of melatonin secretion in ovine pinealocytes* in vitro*.

Although the process of serotonin acetylation is generally considered as the most important step in regulation of melatonin synthesis, the recent studies provided evidence for the rate-limiting role of ASMT in control of the pineal hormone secretion [9, 33]. Moreover, the hypothesis about regulation of melatonin synthesis by interaction of enzymes and regulatory proteins within “melatoninosomes” [26] should also be taken into account in analysis of our data. Therefore, we considered it as important to determine the changes in N-acetylserotonin release from ovine pineal explants during the adrenergic stimulation performed without and with inhibition of translation. For this aim, we developed an HPLC method enabling measurements of N-acetylserotonin and melatonin in the culture medium. The method allowed also an assay of NE at levels used in the* in vitro* studies and precise determination of the temporal relationships between the catecholamine concentration in the medium and the release of indoleamines.

The results obtained in the experiment V showed similar changes in the release of N-acetylserotonin and melatonin during the adrenergic stimulation of ovine pinealocytes and pointed to AA-NAT as a key enzyme in regulation of the pineal hormone secretion in these cells. The data entitled us to conclude that the intensity of serotonin acetylation in ovine pinealocytes is controlled by both protein synthesis independent and dependent phenomena.

Our study showed for the first time that the regulation of N-acetylserotonin synthesis in pinealocytes of some species, like the sheep, involves the mechanism independent of protein synthesis. It provides strong evidence pointing to the need of revision of the current opinion about the control of AA-NAT activity in pinealocytes exclusively by changes in enzyme abundance.

It should be emphasized that the studies of ovine and human AA-NAT* in vitro* and after transfection to nonpineal cells published by Ganguly et al. [29] as well as the crystallographic investigations of AA-NAT structure by Obsil et al. [31] delivered the molecular background for partial explanation of putative mechanisms of the protein synthesis independent regulation of serotonin acetylation. The kinetic analysis of tryptamine and serotonin acetylation by ovine AA-NAT* in vitro* demonstrated tenfold decrease in the arylalkylamine Km after the enzyme phosphorylation at N-terminal portion and binding to 14-3-3 protein [29]. Moreover, the phosphorylation and binding to 14-3-3 protein increased threefold the AA-NAT activity measured using the low (1 *μ*M) serotonin concentration, estimated as corresponding to the physiological level occurring in the ovine pineal gland at night [29]. It is worth mentioning that similar changes in the enzyme affinity in response to the increase in cAMP level were also suggested by the studies performed on 1E7 cell line, which had been obtained by transfection of COS7 cells with wild-type of human AA-NAT [30]. The treatment of these cells with forskolin, an activator of adenylyl cyclase, increased tenfold intensity of melatonin synthesis from 5-methoxytryptamine, without adequate changes in the content of AA-NAT protein [30]. However, immunochemical studies of 1E7 cells suggested that forskolin increased the intracellular conversion of 5-methoxytryptamine to melatonin without changing the phosphorylation state of the N-terminal PKA site in AA-NAT [30]. Also, in the control 1E7 cells, AA-NAT exists with the N-terminal PKA site phosphorylated. Furthermore, in the ovine pineal gland, intensity of immunoreaction with a specific antibody against the AA-NAT phosphorylated at T31 did not differ between day and night [26]. The above presented discrepancies concerning the phosphorylation of N-portion of AA-NAT and its role may suggest that the regulation of enzyme affinity to arylalkylamines and its stability is much more complex than proposed by the current models. The phosphorylation at the C-terminal [34], phosphorylation of other proteins, and formation of large regulatory complexes should be taken into consideration [26]. The influence of cAMP-dependent kinases on AA-NAT may include two phases related to a different affinity of phosphorylation sites in the enzyme molecule or/and in other regulatory molecules, which are responsible for the stabilization of AA-NAT at the first stage and the changes in Km at the second stage. The limited analytical possibilities of antibody-based assay should also be analyzed as a possible source of the abovementioned discrepancies.

The crystallographic analysis demonstrated that the formation of AA-NAT/14-3-3 protein complex modulates the structure of substrate binding sites, enabling the increase in the affinity for arylalkylamines [31]. Moreover, it showed extensive interactions between AA-NAT and 14-3-3 protein, which may be responsible for functioning of intricate regulatory mechanisms. Due to high biological significance, the mechanism through which AA-NAT activity is inhibited or activated requires further, intensive investigations.

The protein synthesis independent activation of melatonin synthesis is hardly detectable in the studies based on measurements of AA-NAT activity in cell or tissue homogenates [19, 30]. The experiments on nonpineal cells transfected with AA-NAT demonstrated that the assay of AA-NAT activity in broken cells did not provide an accurate estimate of intracellular activity of this enzyme [30]. Similarly, in the sheep pineal gland, the contents of N-acetylserotonin and melatonin showed tenfold changes, but AA-NAT activity measured in tissue homogenates demonstrated only three- to fivefold differences [35, 36]. A pulse of light at night induced a rapid decrease in both compounds by more than 85% within 30 min but no significant differences were found in AA-NAT activity during this time. The limitations of AA-NAT activity measurement in cell homogenates may have their sources in several reasons including changes in enzyme properties during homogenization and use of high, nonphysiological concentrations of arylalkylamines.

The second, slow phase of increase in N-acetylserotonin and melatonin release from the ovine pineal explants was dependent on the active translation process and independent of transcription. It is reasonable to consider that it occurs due to inhibition of the AA-NAT proteasomal proteolysis and in consequence the enzyme accumulation in cells. The process of proteasomal proteolysis was initially described in the rat pineal as responsible for the rapid decrease in melatonin secretion in response to light exposition [19]. Later, based on the experiments on bovine pinealocytes, it has been proposed that AA-NAT is continuously produced in these cells and degraded during daytime via the proteasomal proteolysis [20]. The inhibition of proteolysis induced by high cAMP level leads to the increase in AA-NAT level and activity during night [20]. The inhibition of AA-NAT proteolysis has been considered as the main mechanism responsible for the nocturnal elevation of melatonin in many mammalian and nonmammalian species [20, 22, 28, 37]. It seems obvious that AA-NAT molecules accumulated in ovine pinealocytes due to NE-induced inhibition of proteolysis are kept at “high-activity stage.” Huang et al. [38] showed that inhibition of proteasomal proteolysis in cell line stably expressing AA-NAT resulted in a large content of AA-NAT protein but failed to increase the enzyme activity proportional to the amount of proteins accumulated. Recent studies demonstrated that proteasomal proteolysis is also involved in regulation of AA-NAT transcription in rat pinealocytes [39].

Summing up, the intensity of serotonin acetylation in mammalian pinealocytes may be controlled, according to the current knowledge, via three mechanisms: (1) the induction and repression of AA-NAT mRNA transcription, (2) the regulation of proteasomal proteolysis of AA-NAT, and (3) the activation/inactivation of existing AA-NAT molecules. The later process enables rapid changes in melatonin secretion and may play a crucial role in regulation of the pineal activity in many species, including the human. The participation of these mechanisms in generation of the diurnal rhythm of melatonin secretion differs between species. The control of AA-NAT mRNA transcription is the main regulatory mechanism in many rodents and lower vertebrates [17, 37, 40–43], but some prominent exceptions exist: degu [28] and rainbow trout [37]. For comparative purpose, in the present studies, we tested the effect of transcription and translation inhibitors on the release of N-acetylserotonin and melatonin from rat pineals during the adrenergic stimulation. The lack of any increase in release of these indoles from the inhibitor-treated glands demonstrated that the AA-NAT mRNA transcription is an exclusive mechanism controlling the nocturnal increase in pineal secretory activity in this species. The inhibition of proteasomal proteolysis and activation of AA-NAT seem to be the predominating regulatory mechanisms in many mammals including ruminants [20, 22]. The previous study published by Lewczuk [32] suggests that the adrenergic activation of melatonin secretion in the domestic pig occurs exclusively via activation/inactivation of AA-NAT, without involvement of proteins synthesis and degradation. The treatment of pig pineal explants with 10 *μ*M NE resulted in monophasic, rapid, about 5-fold increase in melatonin secretion, which was not affected by translation inhibitors [32]. The adrenergic stimulation had no effect on AA-NAT activity measured using 10 mM of tryptamine, but the increase in activity was noted in the assay performed at 0.1 and 1 mM of this substrate. The regulation of the pineal hormone secretion solely via protein synthesis independent mechanism is probably related to the specific subcellular organization of pig pinealocytes [44, 45] and it is reflected in the diurnal patterns of plasma melatonin level [46, 47].

Our* in vitro* studies provided also interesting data concerning the role of ASMT as a rate-limiting enzyme of melatonin secretion, which has been proposed by Liu and Borjigin [9, 33]. The experiment on rat pineals demonstrated that (1) the N-acetylserotonin release exceeded several folds the melatonin secretion and (2) during the adrenergic stimulation the melatonin release reached the maximum level about one hour before the N-acetylserotonin plateau. The phenomenon that the increase in N-acetylserotonin release is not followed by the subsequent elevation in melatonin secretion is probably caused by the limited efficiency of methylation process. During the initial periods of adrenergic stimulation, the amounts of N-acetylserotonin formed in the pineal gland seem to be below the capacity of ASMT and the release of both amines increases concurrently. When the level of N-acetylserotonin exceeds the efficiency of methylation process, the secretion of melatonin reaches the plateau. The data obtained in our study are in agreement with the result reported using the microdialysis [9, 33]. In contrast to rat pineals, the release of N-acetylserotonin from ovine pineal explants was lower than the melatonin release, both before and during the adrenergic stimulation. However, like in the rat pineals, the increase in N-acetylserotonin release in response to NE-treatment was higher than the elevation of melatonin secretion. Moreover, the pineal hormone achieved the stable level of release before the N-acetylserotonin plateau. The present results demonstrate that ASMT plays an important role in determination of the nocturnal level of melatonin synthesis, but its significance probably differs between species.

## Figures and Tables

**Table 1:** Inhibition of protein synthesis in ovine and rat pineal explants by cycloheximide and anisomycin. Radioactivity of the protein fraction of the pineal explants incubated in the medium containing 0.5% DMSO (control), 100 *μ*M CLX, or 10 *μ*M ANS for 60 minutes and then in the medium containing vehiculum or the inhibitors as well as 10 *μ*M norepinephrine and ^3^H-leucine for 120 minutes. The assay was performed as described in Material and Methods.

Treatment	^ 3^H-leucine incorporation into proteins			
	Ovine pineal explants		Rat pineal explants	
	×10^6^ dpm/mg protein (mean ± SEM, *n* = 6)	%	×10^6^ dpm/mg protein (mean ± SEM, *n* = 6)	%
Control	22.24 ± 1.21	100.00	19.41 ± 0.81	100.00
CLX 100 *μ*M	0.43 ± 0.02	1.92	0.41 ± 0.07	2.11
ANS 10 *μ*M	0.72 ± 0.08	3.21	0.83 ± 0.09	4.22
